# Mental Health Professionals’ Perceptions of Parenting by Service Users with Psychosis

**DOI:** 10.1007/s10597-020-00548-0

**Published:** 2020-01-10

**Authors:** Jennifer Strand, Lisa Rudolfsson

**Affiliations:** 1 Department of Psychology, University of Gothenburg, Box 500, 405 30 Göteborg, Sweden; 2 Gothenburg Research Institute, University of Gothenburg, Box 603, 405 30 Göteborg, Sweden

**Keywords:** Parental psychosis, Parenting interventions, Professionals, Psychosis service, Service needs

## Abstract

Despite extensive needs, interventions for parents with psychosis are rarely offered, poorly described, and vary between offering instrumental and emotional support. To improve the design of interventions offered to families with parental psychosis, more knowledge is needed. The aim of this study was to gain knowledge about mental health professionals’ perceptions of parenting by patients with psychosis. Eleven mental health professionals educated in family interventions were interviewed using a semi-structured interview guide and the material underwent inductive thematic analysis. Results showed that the professionals described the patients parenting as characterized by difficulties in providing security and predictability, taking part in and organizing family life, and to focus on the child’s needs. The difficulties were described as related to specific symptoms such as voice hearing, cognitive impairments, anxiety, and paranoia. As a vast amount of research stresses the psychosocial basis of psychosis and the interpersonal causes of its symptoms, parenting difficulties in people with psychosis could benefit from being addressed from a relational perspective. Accordingly, parents with psychosis should be offered interventions that enable them to create positive parental role models, develop reflective functioning, and identify situations in which their symptoms might hinder positive parenting. Many of these needs are unmet by interventions offered in adult psychosis services today.

## Introduction

Psychotic disorders are serious mental illnesses affecting approximately 0.5–1.5% of the general population (McGrath et al. 2004). Among the many symptoms are paranoid ideations, hallucinations, lethargy, and withdrawal (American Psychiatric Association 2013). Cognitive impairment is also prevalent among individuals suffering from psychosis, in particular among people suffering from schizophrenia (Heinrichs and Zakzanis 1998).

It is well known that children of parents with psychosis are at high risk of developing mental illness themselves. Having one parent diagnosed with schizophrenia results in a 7% increased lifetime risk of schizophrenia and a 55% increased risk of developing any psychiatric condition (Gottesman et al. 2010). Moreover, compared to children of parents without known mental illness, children of parents diagnosed with schizophrenia tend to display more cognitive (Welham et al. 2009) and emotional problems (Niemi et al. 2005), attention difficulties, and poorer social adjustment at school (Erlenmeyer-Kimling et al. 2000). There is also a significantly higher risk for these children than for children of parents with bipolar disorders and major depressive disorders to develop mood disorders (Rasic et al. 2014).

Considering current research showing that children of parents with psychosis are at risk of developing mental illness themselves (Gottesman et al. 2010), the support needs of parents needs to be identified. A previous systematic review showed that mothers with severe mental illness are challenged in their parenting by guilt, isolation, and stigma (Dolman et al. 2013). Another study showed that many parents described feeling unable to cope as a parent, feeling isolated because of symptoms such as paranoia, having meagre financial resources, and feeling guilty about exposing their children to their symptoms or to genetic risks (Schrank et al. 2015). Parents with mental illness also often have low educational attainment, unemployment, and poverty, which affect the chances of the child’s practical and social needs being met (Campbell et al. 2012). Symptoms such as delusions, hallucinations, cognitive impairment, and fatigue can also affect parents’ attention, communication, and ability to meet the child’s emotional needs (Healy et al. 2016; Kahl and Jungbauer 2014). As a result of symptoms and guilt, some parents can be unaware of how their illness affects their parenting, and the child’s everyday life and development (Pihkala et al. 2012). However, it is important to note although a majority of studies investigating parenting in people of psychosis show that many parents are in need of support, a recent study suggest that a majority of parents were rated as good-enough or adequate parents in terms of their participation, interest, and competence in childcare (Campbell et al. 2018).

Interventions that target parenting in people with psychosis are rarely offered despite the extensive parental needs. Two review studies (Gearing et al. 2012; Schrank et al. 2015) showed that no interventions have been designed specifically for parents with psychosis and that more knowledge about these parent’s difficulties and needs are important. Although the parent’s subjective understanding may offer the most in-depth understanding of the situation in families with parental psychosis, there can be barriers to involving them in research studies. For example, many parents’ fear that child protection services may become involved and, further, they can be reluctant to acknowledge the possible adverse effects of their illness on their children out of fear of stigma (Maybery and Reupert 2009). Accordingly, parenting by people with psychosis could benefit from being studied from several perspectives. The perceptions of mental health professionals are one perspective that could offer additional information to the parents own understanding, partly because of their experiences of providing care to the parents and partly because they conduct interventions with the service users’ families. In Sweden, the professionals with the most insight in the families of their service users are social workers and psychiatric aides. These professionals are also the ones who has received training in family interventions, offered to parents with psychosis. Therefore, the aim of this study was to gain knowledge about these professionals’ perceptions of parenting by service users with psychosis. However, it is important to emphasise that the professionals experiences may primarily be based on families in need of support and do not reflect all families where a parent has psychosis.

## Method

This study is part of a larger research project called *Evaluating Parent-Based Interventions Targeting Children of Parents with Psychosis.* The research project was carried out at nine psychiatric outpatient clinics for service users with psychosis. The units were located in diverse areas of Sweden, representing low to high socioeconomic statuses. One part of the research project concerns mental health professionals’ experiences of supporting parenting and integrating the child’s perspective into adult psychosis service. The design of the research project was approved by the Regional Ethical Review Board, University of Gothenburg (ref. no. 599-15).

### Participants

Eleven women, aged 32 to 57 years, participated in the study. Nine units were included in the research project, but only eight are represented in this study as no mental health professional from one of the units chose to participate.

Participants’ work experience ranged from three to 26 years (M = 13.09). Six participants worked as social workers, four as psychiatric aides, and one as a licensed nurse. All participants were responsible for making sure that the child perspective permeated their organization and they had all been educated in Beardslee’s Family Intervention (FI; Beardslee et al. 2003) and/or Let’s Talk About the Children (LTC; Solantus 2006). Both interventions are manual-based secondary prevention programmes aimed to provide information about how the parent’s mental illness can affect the child and to promote communication about the parents’ illness in the family.

### Procedure

The unit heads at each of the nine participating outpatient clinics were asked to send a list with names and contact information for all staff who were trained in FI and/or LTC. An invitation to participate in the study was sent by email to all trained professionals describing the aim of the study, the voluntary nature of participation, and the guarantee of anonymity. Of the 37 professionals trained in FI and/or LTC, seven initially agreed to participate. A reminder email sent one month later to non-responders prompted another three professionals to accept, and a last reminder sent another month later recruited one more participant, for a total of 11.

Interviews were scheduled within two weeks of the professionals’ agreement to participate. One interview was conducted at the Department of Psychology, University of Gothenburg; all others were carried out at the professionals’ respective psychiatric units.

### Interview

The interview guide was semi-structured and covered two main areas: (1) the mental health professionals’ perceptions of the families’ situations and needs and (2) the mental health professionals’ experiences of conducting family interventions. This article offers findings about mental health professionals’ perceptions of parenting by service users with psychosis. The mental health professionals’ experiences of conducting family interventions, (i.e. FI and LTC), has been published in another paper (ref will be inserted).

All interview questions were open-ended and participants were asked to speak partly out of their general understanding and partly in relation to specific families they had met. Examples of questions are: If you were to describe the situation in a typical family that you meet, how would you describe that? Do you know if your service user’s mental illness affects their parenting, if so, how? Are there any specific symptoms that you perceive to affect parenting more than others? What perception do you have about what parenting support your service users need? Follow-up questions were used to clarify participants’ experiences and thoughts and varied among interviews, depending on how forthcoming and detailed the participant was.

The interviews each lasted approximately one hour. The first author conducted six interviews, and the second author five. All interviews were audio-recorded and transcribed verbatim. To become familiar with all interviews, each author transcribed the other’s interviews.

### Analysis

The transcripts were analysed using inductive thematic analysis, which can be described as data-driven or bottom-up (Braun and Clarke 2006). The transcripts were first read by both authors to extract all data relevant to the research question (i.e., the professionals’ perceptions of parenting abilities in parents with psychosis). The data were then coded separately by both authors, who made no attempt to fit data into a pre-existing framework. Ideas for structuring the data were noted. All codes and ideas were then discussed by the authors and new ideas were used in re-coding. With a focus on professionals’ understanding of parental abilities, the codes were first organized into subthemes according to the difficulties described and the effect of these difficulties on the children. The coded data extracts were then reorganized into two main themes that captured the professionals’ views of parenting, their associations with symptoms of psychosis, and its effect on the children. Finally, all data extracts were reviewed to find quotations that best captured the essence of each main theme and subtheme. Participants have been given pseudonyms to respect their confidentiality.

## Results

Results are based on the professionals’ perceptions of their service users’ parenting and its effect on their children. All professionals stressed that almost all parents struggled to make sure that their children’s emotional and physical needs were met. Yet, the main focus in the interviews became the service users’ difficulties with practising aspects of positive parenting such as proving security and comfort. Therefore, the professionals’ perceptions of parenting by service users with psychosis are structured along two main themes: (1) parents’ difficulty to provide security and predictability, and (2) parents’ difficulty to provide emotional guidance and comfort. Table 1 shows main and subthemes.

**Table 1:** Main and subthemes

Main theme	Subtheme
Parent’s difficulty to provide security and predictability	A frightened parent An unorganized parent
Parent’s difficulty to provide emotional guidance and comfort	A parent who is occupied with their own emotions The child taking on the role of the parent

### Parents’ Difficulty to Provide Security and Predictability

The professionals commonly described how some parents’ difficulties to provide their children with security was a consequence of the parent her/himself being frightened, often as a consequence of paranoia. The inability to provide the child with predictability was commonly attributed to parents lacking the ability to organize and plan family life.

#### A Frightened Parent

All professionals described the difficulties when their service users suffered from paranoia and how that could have implications for their abilities to parent. A general understanding was that symptoms of paranoia could make the parent vigilant, suspicious, and stressed. Oftentimes, the service users were also described as lacking insight in how such parenting behaviour affected their children.

I come to think of this mother we meet. It was very hard during the family meeting, because she was very angry at everything, and you could understand that she lacked the ability to comprehend how her anger affected her children, that she scares them. (Isa).

Many professionals speculated about how parents with paranoid ideations could overreact to internal and external cues of potential danger, which could create a parent who was unable to provide security, especially when the parents’ emotional reaction invoked irrational and frightening behaviour. One of the professionals described a mother who believed that the colour black meant that a family member was going to die.

I remember one family, where the mother was terrified by the colour black; the boy was not allowed to wear black clothes when the mother was ill. In such cases, you come to think “My God, what has this child been told?” Now they’re able to talk about it and laugh at it, but still… (Fiona).

Some professionals described parents whose suspicion was directed towards the child. Such beliefs were thought to make the parent both frightened and sometimes even frightening, especially for younger children who rely upon the parent’s reactions to external stimuli.

Some parents develop fears about their children. There are some symptoms that involve fear of the child being injured, and that will make the child afraid, too. (Anne).

According to the professionals, paranoia involving beliefs about the child being in danger led many parents to become overprotective and restrictive of the child. They gave examples of parents who, out of worry or suspicion, made phone calls to school to make sure that the child was well or who did not allow their children to drink water from the tap. Some professionals also described how parent’s fear and suspicion could make it hard for them to participate in social activities, affecting the child’s ability to, for example, get involved in a sports activity.

The fear and suspicion, all the worries of interacting with others. The perceived danger of being in a room with other parents at PTA meetings and sports activities. Some children won’t get the chance to get out of their homes if they don’t have anyone else to take them. (Johanna).

#### An Unorganized Parent

A reoccurring theme was how cognitive impairments, caused either by the illness or by medications, caused some parents difficulty in attaining insight into their illness and in structuring family life. Many professionals stressed that parents who lacked understanding of their symptoms, were less able to protect their children from their symptoms. Some particularly emphasized the importance of parents’ cognitive capacity, such as insight, when they explained their illness experiences to their child.

Many parents lack insight into their illness; they try not to think about their illness and lack self-reflection. Of course, that makes it hard to support their children, and things can go very wrong when they try to explain their way of being to the child. (Bea).

Difficulties in planning and structuring family life were described as another obstacle to parenting. These difficulties were thought to reduce parents’ capacities to maintain daily routines such as planning and cooking meals, making sure that school work is done, or planning the child’s leisure activities.

When patients are more ill, they often struggle with planning, which can affect the child quite concrete; the child is not dressed properly, and no one buys or prepares food for them. It’s a big difference between having a parent who is cognitively impaired and having a parent who has the flu. (Dina).

Symptoms such as fatigue and reduced motivation was described as causing distress for the family, and seen as potentially making the parent unable to structure family life. A general understanding was that some parents, in periods, were unable to take part in, or carry out, daily activities and routines.

…when you don’t have the power or ability to even get out of bed in the morning. Those parents lack the ability to get things done, they spend all day on their sofa, and of course, that creates anxiety for the child. (Isa).

However, a few professionals also described how negative symptoms such as emotional numbness and extreme tiredness, might be easier for the child to understand since a parent with negative symptoms behaves more as a physically ill person is expected to (i.e., stays in bed or does not shower). They also speculated that negative symptoms could lead to more obvious child neglect, which is easier for adults outside the family to detect.

Negative symptoms are often more apparent to the children and to those in their surroundings. For example, the school may notice that the child is not appropriately dressed for the season, and then they might get more support. (Fiona).

### Parents’ Difficulty to Provide Emotional Guidance and Comfort

This theme concerns the professionals’ descriptions of some parents’ difficulties in setting aside their own anxiety and needs to focus on the child’s needs for comfort and emotional guidance. The professionals commonly described how some parents’ difficulties to support and comfort their children was a consequence of the parent her/himself lacking the ability to identify and regulate emotions, thus, making it hard to understand their children’s emotional expressions. Consequently, the children of parents with psychosis were described as having to withhold their own emotional needs, and in some cases take on the role of the parent themselves.

#### A Parent Who is Occupied with Their Own Emotions

Some professionals described parents who had difficulties expressing their emotions. In these families, the emotional climate was perceived as “meagre”, and some talked about parents who were too anxious or unable to show affection towards their children. Other parents were perceived as having difficulty regulating their emotions, described as leading to intense expressions of some feelings, particularly fear and aggression. All professionals described parents who had emotional difficulties and either minimized or maximized their own emotional needs. In either case, their lack of recognition about how their own emotional state might affect their children was described as an obstacle to the parents’ ability to guide and support the child. Some children were described as being forced to focus on their parent’s emotions, rather than their own.

The children aren’t given the tools to express their own emotions since the parent isn’t able to. And they are forced to shift their focus; they are focused on their parent rather than on practising to handle their own emotions. (Bea).

The parents’ emotional difficulties were explained not only as a result of their illness, but also as a consequence of their own childhood experiences; the professionals emphasized that many of these parents had themselves grown up in dysfunctional families with violence and abuse. These experiences were described as having left the parents without role models. Some professionals were also concerned that these problems would be passed on to the next generation.

Many of our patients weren’t taught to manage their emotions when they themselves were children, and how are they then to be able to provide warmth, security, and love to their children? Although they do love their children. Well, these difficulties with expressing emotions, we see it across generations; and so why would it stop with our patients and their children? (Gisela).

Although the professionals described their service users as loving their children, they also described how some were unable to show such affection or to offer comfort. Some parents were described as being so focused on their own distress, anxiety, and/or voices that the child’s need for comfort was obscured.

Some parents can’t tell if their child is sad, [because] they’re so occupied with their own sadness. Then the child won’t get any comfort, since their parent can’t understand that that is what they need; the parent is unable to meet their child’s needs because they’re occupied by their own needs. (Anne).

One professional talked about a family in which comfort had become the other parent’s task, and described how the child sometimes had to postpone his emotional needs until the other parent was present.

I think that some children have to wait for comfort. I had one mother who told me that when the child hurt himself, he had to wait to be comforted until his father came home. (Johanna).

Some professionals emphasized that the parents’ difficulties became more acute when their symptoms became more severe. One of the professionals exemplified the inability of some parents to offer emotional guidance and comfort by describing a mother who became so occupied by her voices that her daughter’s most basic needs were unmet. Consequently, some children were described as growing up in an emotional environment that required them to minimize their own needs.

I think that many of those children hold back on their own needs in favour of their ill parent. (Eva).

#### The Child Taking on the Role of the Parent

Some parents were described as relying on their children when they themselves were distressed, anxious, or in need of company. Such behaviour was commonly explained by the parent’s loneliness or difficulty with setting their own needs aside. As a consequence of the parents inability to organize and plan family life, as well as their inability to regulate their own emotions many professionals described children to parents with psychosis as forced to take on a greater responsibility than a child should need to.

The children are often forced to take on more responsibility than they are supposed to. (Hanna).

Some professionals gave examples of when parents had used the child for comfort and confirmation, especially at times when they were more ill.

I meet one mother who treats her child as an adult. She talks about her own needs, and I feel like she has a hard time seeing her child and caring for it, and of course, when she gets ill it becomes even harder. (Gisela).

As a strategy to cope with their own anxiety, some parents were described as using their children for comfort, which could cause the other parent concern and sometimes lead to conflicts between the parents.

The father accuses the mother of letting their son sleep in the same bed as her; he thinks that it’s intrusive and shows a lack of boundaries. I’m not sure, but perhaps she wants their son in her bed to give her comfort and security. (Cecilia).

## Discussion

Even though all professionals stressed that the parents loved their children, the interviews became focused on the negative aspects of being a parent with psychosis. These negative descriptions were characterised by difficulties providing security and predictability, commonly attributed to symptoms of paranoia and lacking the ability to organize family life, and difficulties focusing on the child’s needs, commonly attributed to the parent being occupied with their own emotions. These difficulties were described as resulting in the child not being given emotional support and as making the child take on a greater responsibility than they should need to; sometimes forcing the child to take on the role of the parent.

The results will be discussed according to the difficulties described by professionals, and the support needed to address these difficulties and assist parents with psychosis to develop more positive parenting abilities.

### Parents’ Fear and Lack of Positive Role Models

The professionals described paranoia as one of the most influential symptoms on parenting, however not the only one. Symptoms of paranoia was described as making the parent vigilant, suspicious, and stressed. Paranoid ideations was also described as potentially making the parent overreact to internal and external cues of potential danger; creating a parent who was unable to provide security. When suspicion was directed towards the child, parents were described as being both frightened and sometimes even frightening, especially for younger children who rely upon the parent’s reactions to external stimuli. Oftentimes, the service users was also described as lacking insight in how such parenting behaviour affected their children. Parents who lacked understanding of their symptoms, were described as less able to protect their children from their symptoms. The importance of parents’ cognitive capacity, such as insight, was stressed as important when parents explained their illness experiences to their child.

Paranoia is, by definition, a problem of interpersonal reference, with excessive attention to the behaviours and feelings of others. Moreover, a lack of social identification or belongingness underlies paranoia (Freeman et al. 2008), and a recent large-scale study shows that enhancing social identification reduces its symptoms (McIntyre et al. 2017). Considering the interpersonal roots of paranoia, it is not surprising that this symptom has implications for caregiving. In line with previous research that suggests that social identification might reduce paranoia (McIntyre et al. 2017), parenting interventions could be conducted in groups. There are also studies showing that the service users themselves request groups focused on both general parenting and parenting while experiencing psychosis (Alakus et al. 2007). Such groups might provide a safe social context in which the participants can share their experiences and fears. Parenting interventions could also offer participants the opportunity to reflect upon and share their experiences of how interpersonal insecurity influences their caregiving and parenting; it could also help parents identify situations in which their fear restricts their child’s need to explore.

The professionals emphasized that many parents had grown up in dysfunctional families with violence and abuse, and they attributed some parents’ inability to comfort and show emotional warmth to their family of origin. Such experiences were also described as having left the parents without role models. These accounts are supported by several studies showing associations between psychosis and childhood adversities such as sexual, physical, and emotional abuse (e.g., Varese et al. 2012; Bonoldi et al. 2013). Qualitative studies also report descriptions of service users’ own parents as abusive and uncaring and their family of origin’s emotional climate as cold, meagre, and silent (e.g., Strand and Tidefors 2012).

Role models are an important aspect of caregiving and parenting. It is well-known that parenting and behaviours are based on experience (i.e., influenced by experiences with the parents own parents). It is also known that individuals with experiences of abuse in their family of origin struggle with more parenting anxiety, stress, and insecurity than others (Hugill et al. 2017). An important part of interventions for parents with negative experiences in their family of origin is to help parents choose positive role models to emulate in their parenting. Focusing on how parents’ experiences of caregiving in their family of origin influences their own parenting could also be a way to help them reflect upon and identify parental strategies based on more positive role models. An experience-based intervention focused on the link between past and present relations, instead of the link between the illness and the shortcomings of the parents, might be perceived as less stigmatizing and attract service users to take part in family interventions; a task that numerous studies has proven to be difficult (Schrank et al. 2015).

### Parents’ Difficulties in Recognizing and Responding to the Child’s Needs and Feelings

The professionals described parents’ difficulties in setting aside their own anxiety and needs to focus on the child’s needs for comfort and emotional guidance, and the emotional climate in such families was described as meagre. Some parents were described as too anxious or as unable to show affection towards their children, while other parents were described as having difficulties regulating their own emotions which could lead to intense expressions of particularly fear and aggression. Parents’ lack of recognition about how their own emotional state might affect their children was described as an obstacle to the parents’ ability to guide and support the child and some children were described as being forced to focus on their parent’s emotions, and as sometimes taking on the role of the parent.

The ability to make sense of and adequately respond to both their own and others’ behaviours, needs, and feelings is referred to as mentalization (Fonagy and Target 2006). Problems in mentalization are common in people with schizophrenia (Sprong et al. 2007). A concept related to mentalization, but used to describe parents’ ability to understand the child’s mental states, is parental reflective functioning (RF). A recent review of studies in non-clinical samples shows that higher parental RF is associated with adequate caregiving, while low parental RF is found in mothers whose children suffer from anxiety, impairment in emotion regulation, and externalizing behaviours (Camoirano 2017). There is also scientific support for an association between a history of abuse and neglect and lower parental RF (Berthelot et al. 2015). Clinical interventions designed to improve parental RF could usefully be offered to parents with psychosis for several reasons: the strong associations between schizophrenia and low levels of mentalization (Schiffman et al. 2004; Sprong et al. 2007); parents’ difficulties in recognizing and coping with the child’s needs and emotions (Healy et al. 2016); and the negative affect of neglect and abuse on parental RF (Berthelot et al. 2015).

Some parents were described as relying on their children when they themselves were distressed, anxious, or in need of company. Moreover, because of some parents’ inability to organize and plan family life, many professionals described children who had to take on a greater responsibility than a child should need to. Children taking on parental responsibilities to an extent that exceeds the developmental norms in the culture is sometimes referred as role reversal (e.g., Boszormenyi-Nagy and Spark 1973). Responsibilities can include giving the parent instrumental help (e.g., cooking, cleaning, looking after younger siblings) and emotional help (e.g., advice, comfort, reassurance, company). A more recent, child-focused perspective is the concept of “young carers”, i.e., children and youths who provide practical and/or emotional support to a family member who has any type of illness (Dearden and Becker 1997). The professionals in this study confirmed that some children provided both instrumental and emotional help to their ill parent. Instrumental help was most commonly described as a response to the parent lacking motivation, and emotional help was described as related to parental anxiety.

From a relational perspective (Boszormenyi-Nagy and Spark 1973), role reversal stems from the parent’s unsatisfied needs to be parented and cared for. From an attachment perspective (Bowlby 1980), the parent is seen as unable or unwilling to give the child the protection, support, and the care it needs presumably because the parent’s own needs for reassurance and protection remain unmet. Based on our findings, a combination of both theories could enrich the understanding of role reversal because these parents appear to have both unsatisfied needs of being cared for and extensive needs for reassurance and protection because of their anxiety and fear.

In individual therapy, role reversal is often treated by enhancing the parent’s own sense of security (Byng-Hall 2002). Parenting interventions could more appropriately focus on exploring and providing information about children’s emotional needs and the possible consequences if their needs are unmet. Research using the concept of young carers also shows the importance of incorporating the family system and providing instrumental family support to unburden the child (Wahl et al. 2017). More research focusing on the underlying mechanisms of role reversal in this group of parents is necessary. A better understanding could also address role reversal in parenting interventions for service users with psychosis.

### Clinical Implications

In addition to psychological and psychopharmacological treatments, the results indicate that interventions targeting parenting in patients with psychosis could preferably be held in groups and include elements of (1) linking childhood experiences of caregiving to their own parenting, (2) creating positive role models to use as a basis for parenting, (3) developing parental RF, (4) educating parents about their children’s emotional needs and attachment signals, (5) and identifying situations in which symptoms hinder satisfactory parenting. The psycho-educative elements found in existing family interventions, such as BFI and FI, are also an important aspect in supporting parents with psychosis and their children.

### Limitations and Areas of Further Studies

It is important to stress that these findings are based on the professionals’ perceptions and should not be assumed to accurately describe any actual family situation. However, the professionals’ had extensive experience in working with these patients and their families and through their role as child coordinators at the service units. It is also important to emphasize that although the interview questions were open-ended and focused on parenting abilities, the professionals mainly discussed the parents’ difficulties and problems. Therefore, it is important to note that other studies have shown greater heterogeneity in the parenting quality of parents with psychosis (i.e. Campbell et al. 2018). It is also possible that mental health professionals are less reluctant to report adversities than the parents themselves. Another possibility is that mental health professionals are more focused on attending to difficulties and needs. However, the discrepancies between the perceptions of mental health professionals and those of the parents themselves require further studies.

## References

[CR1] Alakus C, Conwell R, Gilbert M, Buist A, Castle D (2007). The needs of parents with a mental illness who have young children: An Australian perspective on service delivery options. International Journal of Social Psychiatry.

[CR2] American Psychiatric Association (2013). Diagnostic and statistical manual of mental disorders.

[CR3] Beardslee WR, Gladstone TR, Wright EJ, Cooper AB (2003). A family-based approach to the prevention of depressive symptoms in children at risk: Evidence of parental and child change. Pediatrics.

[CR4] Berthelot N, Ensink K, Bernazzani O, Normandin L, Luyten P, Fonagy P (2015). Intergenerational transmission of attachment in abused and neglected mothers: The role of trauma-specific reflective functioning. Infant Mental Health Journal.

[CR100] Bonoldi I, Simeone E, Rocchetti M, Codjoe L, Rossi G, Gambi F, Fusar-Poli P (2013). Prevalence of self-reported childhood abuse in psychosis: A meta-analysis of retrospective studies. Psychiatry Research.

[CR5] Boszormenyi-Nagy I, Spark G (1973). Invisible loyalties: Reciprocity in intergenerational family therapy.

[CR6] Bowlby J (1980). Attachment and loss: Loss, sadness and depression.

[CR7] Braun V, Clarke V (2006). Using thematic analysis in psychology. Qualitative Research in Psychology.

[CR8] Byng-Hall J (2002). Relieving parentified children’s burdens in families with insecure attachment patterns. Family Process.

[CR9] Camoirano A (2017). Mentalizing makes parenting work: A review about parental reflective functioning and clinical interventions to improve it. Frontiers in Psychology.

[CR10] Campbell L, Hanlon M, Poon AWC, Paolini S, Stone M, Galletly C, Cohen M (2012). The experiences of Australian parents with psychosis: The second Australian national survey of psychosis. Australian and New Zealand Journal of Psychiatry.

[CR11] Campbell, L. E., Hanlon, M. C., Galletly, C. A., Harvey, C., Stain, H., Cohen, M., … Brown, S. (2018). Severity of illness and adaptive functioning predict quality of care of children among parents with psychosis: A confirmatory factor analysis. *Australian and New Zealand Journal of Psychiatry*, *52*, 435–445. 10.1177/0004867417731526 29103308

[CR12] Dearden C, Becker S (1997). Protecting young carers: Legislative tensions and opportunities in Britain. Journal of Social Welfare and Family Law.

[CR13] Dolman C, Jones I, Howard LM (2013). Pre-conception to parenting: A systematic review and meta-synthesis of the qualitative literature on motherhood for women with severe mental illness. Archives of Women’s Mental Health.

[CR14] Erlenmeyer-Kimling L, Rock D, Roberts SA, Janal M, Kestenbaum C, Cornblatt B, Gottesman II (2000). Attention, memory, and motor skills as childhood predictors of schizophrenia-related psychoses: The New York High-Risk Project. The American Journal of Psychiatry.

[CR15] Fonagy P, Target M (2006). The mentalization-focused approach to self-pathology. Journal of Personality Disorders.

[CR16] Freeman D, Pugh K, Antley A, Slater M, Bebbington P, Gittins M, Garety P (2008). Virtual reality study of paranoid thinking in the general population. The British Journal of Psychiatry.

[CR17] Gearing R, Alonzo D, Marinelli C (2012). Maternal schizophrenia: Psychosocial treatment for mothers and their children. Clinical Schizophrenia & Related Psychoses.

[CR18] Gottesman II, Laursen TM, Bertelsen A, Mortensen PB (2010). Severe mental disorders in offspring with 2 psychiatrically ill parents. Archives of General Psychiatry.

[CR20] Healy SJ, Lewin J, Butler S, Vaillancourt K, Seth-Smith F (2016). Affect recognition and the quality of mother-infant interaction: Understanding parenting difficulties in mothers with schizophrenia. Archives of Women's Mental Health.

[CR21] Heinrichs RW, Zakzanis KK (1998). Neurocognitive deficit in schizophrenia: A quantitative review of the evidence. Neuropsychology.

[CR101] Hugill M, Berry K, Fletcher I (2017). The association between historical childhood sexual abuse and later parenting stress: A systematic review. Archives of Women's Mental Health.

[CR22] Kahl Y, Jungbauer J (2014). Challenges and coping strategies of children with parents affected by schizophrenia: Results from an in-depth interview study. Child & Adolescent Social Work Journal.

[CR26] Maybery D, Reupert A (2009). Parental mental illness: A review of barriers and issues for working with families and children. Journal of Psychiatric and Mental Health Nursing.

[CR27] McGrath J, Saha S, Welham J, El Saadi O, MacCauley C, Chant D (2004). A systematic review of the incidence of schizophrenia: the distribution of rates and the influence of sex, urbanicity, migrant status and methodology. BMC Medicine.

[CR28] McIntyre J, Wickham S, Barr B, Bentall R (2017). Social identity and psychosis: Associations and psychological mechanisms. Schizophrenia Bulletin.

[CR29] Niemi LT, Suvisaari JM, Haukka JK, Lönnqvist JK (2005). Childhood predictors of future psychiatric morbidity in offspring of mothers with psychotic disorder: Results from the Helsinki High-Risk Study. The British Journal of Psychiatry.

[CR30] Pihkala H, Sandlund M, Cederström A (2012). Initiating communication about parental mental illness in families: An issue of confidence and security. International Journal of Social Psychiatry.

[CR31] Rasic D, Hajek T, Alda M, Uher R (2014). Risk of mental illness in offspring of parents with schizophrenia, bipolar disorder, and major depressive disorder: A meta-analysis of family high-risk studies. Schizophrenia Bulletin.

[CR102] Schiffman J, Lam CW, Jiwatram T, Ekstrom M, Sorensen H, Mednick S (2004). Perspective-taking deficits in people with schizophrenia spectrum disorders: A prospective investigation. Psychological Medicine.

[CR32] Schrank B, Moran K, Borghi C, Priebe S (2015). How to support patients with severe mental illness in their parenting role with children aged over 1 year? A systematic review of interventions. Social Psychiatry and Psychiatric Epidemiology.

[CR34] Solantus T (2006). Föra barnen på tal: När en förälder har psykisk ohälsa. (Let’s talk about the children: When a parent suffers from mental illness).

[CR35] Sprong M, Schothorst P, Vos E, Hox J, van Engeland H (2007). Theory of mind in schizophrenia. The British Journal of Psychiatry.

[CR36] Strand J, Tidefors I (2012). “If you’re not safe anywhere, you turn it inside yourself”: Narratives about childhood experiences told by 12 individuals diagnosed with psychosis. Psychosis.

[CR38] Varese F, Smeets F, Drukker M, Lieverse R, Lataster T, Viechtbauer W, Bentall R (2012). Childhood adversities increase the risk of psychosis: A meta-analysis of patient-control, prospective- and cross-sectional cohort studies. Schizophrenia Bulletin.

[CR39] Wahl P, Bruland D, Bauer U, Okan O, Lenz A (2017). What are the family needs when a parent has mental health problems? Evidence from a systematic literature review. Journal of Child and Adolescent Psychiatric Nursing.

[CR40] Welham J, Scott J, Williams G, Najman J, Bor W, O’Callaghan M, McGrath J (2009). Emotional and behavioural antecedents of young adults who screen positive for non-affective psychosis: A 21-year birth cohort study. Psychological Medicine.

